# Distinct genotypic profiles of the two major clades of *Mycobacterium africanum*

**DOI:** 10.1186/1471-2334-10-80

**Published:** 2010-03-29

**Authors:** Sidra E Gonçalves Vasconcellos, Richard C Huard, Stefan Niemann, Kristin Kremer, Adalberto R Santos, Philip N Suffys, John L Ho

**Affiliations:** 1 Laboratory of Molecular Biology Applied to Mycobacteria, Oswaldo Cruz Institute, Oswaldo Cruz Foundation, Avenida Brasil 4365, Manguinhos - 21040-900, Rio de Janeiro, Brazil; 2 Clinical Microbiology Service and the Department of Pathology, New York-Presbyterian Hospital, Columbia University Medical Center, 622 West 168th Street, New York City, NY, USA; 3 National Reference Center for Mycobacteria, Forschungszentrum, Parkallee 18, D-23845, Borstel, Germany; 4 Mycobacteria Reference Laboratory, (CIb/LIS), National Institute for Public Health and the Environment, 3720 Bilthoven, the Netherlands; 5 Division of International Medicine and Infectious Diseases, Department of Medicine, Joan and Sanford I. Weill Medical College of Cornell University, 1300 York Avenue, New York City, NY, USA

## Abstract

**Background:**

*Mycobacterium tuberculosis *is the principal etiologic agent of human tuberculosis (TB) and a member of the *M. tuberculosis *complex (MTC). Additional MTC species that cause TB in humans and other mammals include *Mycobacterium africanum *and *Mycobacterium bovis*. One result of studies interrogating recently identified MTC phylogenetic markers has been the recognition of at least two distinct lineages of *M. africanum*, known as West African-1 and West African-2.

**Methods:**

We screened a blinded non-random set of MTC strains isolated from TB patients in Ghana (*n *= 47) for known chromosomal region-of-difference (RD) loci and single nucleotide polymorphisms (SNPs). A MTC PCR-typing panel, single-target standard PCR, multi-primer PCR, PCR-restriction fragment analysis, and sequence analysis of amplified products were among the methods utilized for the comparative evaluation of targets and identification systems. The MTC distributions of novel SNPs were characterized in the both the Ghana collection and two other diverse collections of MTC strains (*n *= 175 in total).

**Results:**

The utility of various polymorphisms as species-, lineage-, and sublineage-defining phylogenetic markers for *M. africanum *was determined. Novel SNPs were also identified and found to be specific to either *M. africanum *West African-1 (*Rv1332*^523^; *n *= 32) or *M. africanum *West African-2 (*nat*^751^; *n *= 27). In the final analysis, a strain identification approach that combined multi-primer PCR targeting of the RD loci RD9, RD10, and RD702 was the most simple, straight-forward, and definitive means of distinguishing the two clades of *M. africanum *from one another and from other MTC species.

**Conclusion:**

With this study, we have organized a series of consistent phylogenetically-relevant markers for each of the distinct MTC lineages that share the *M. africanum *designation. A differential distribution of each *M. africanum *clade in Western Africa is described.

## Background

Mycobacteria that cause human and/or animal tuberculsosis (TB) are grouped together within the *Mycobacterium tuberculosis *complex (MTC). The MTC is comprised of the classical species *M. tuberculosis*, *Mycobacterium africanum*, *Mycobacterium microti*, and *Mycobacterium bovis *(along with the widely used vaccine strain *M. bovis *bacillus Calmette-Guérin [BCG]) [1-3], as well as newly recognized additions *Mycobacterium caprae *and *Mycobacterium pinnipedii *[4,5]. Although they are not presently officially described microorganisms, "*Mycobacterium canettii*" (proposed name), the oryx bacillus, and the dassie bacillus are additional widely-accepted members of the MTC [6-8]. *M. tuberculosis *is the predominant cause of human TB worldwide but *M. africanum *and *M. bovis *remain important agents of human disease in certain geographical regions. Of note, *M. bovis *is naturally resistant to pyrazinamide, a first-line anti-TB drug [9], and so treatment of human TB caused by *M. bovis *should not include pyrazinamide. Therefore, the correct identification of MTC isolates to the species level is important to ensure appropriate patient treatment, as well as for the collection of epidemiological information and for implementing necessary public health interventions.

Mycobacteriological laboratory methods have traditionally utilized a series of tests based upon growth, microscopic, phenotypic, and biochemical properties in order to segregate the classical members of the MTC [10]. However, these tests can be slow-to-results, cumbersome, imprecise, non-reproducible, time-consuming, may not give an unambiguous result in every case, and may not be performed by every clinical microbiology laboratory. The relatively recent identification of DNA sequence level differences amongst the species of the MTC has greatly improved our capacity for performing molecular epidemiology, phlylogenetic structuring of the MTC evolutionary tree, and MTC species determination. Molecular techniques, such as PCR, either alone or followed by sequence analysis or restriction fragment analysis (RFA), have proven particularly useful for the characterization of single nucleotide polymorphisms (SNPs) and/or chromosomal region-of-difference (RD) loci (such as insertions, deletions, and rearrangements) that are either lineage-, species-, or strain-specific [7]. Several groups have reported on the development of molecular protocols for the definitive identification of unknown MTC isolates to the species level by RD and/or SNP analysis [2,7,11-13] and clinical laboratories are now beginning to integrate such home-brew protocols into their routine identification protocols for acid-fast bacilli. The only currently available commercial protocol for MTC species identification is the GenoType MTBC^® ^assay (Hain Lifescience, Nehren, Germany) that can differentiate *M. tuberculosis *from *M. africanum*, *M. microti*, *M. caprae*, *M. bovis*, and *M. bovis *BCG [14-16]. However, this test is limited in that it cannot differentiate all species of the MTC and it is not commercially available for diagnostic purposes in the USA.

In the past, *M. africanum *strains were generally identified by default, having first ruled-out both *M. tuberculosis *and *M. bovis *by the traditional battery of tests. Two biovars of *M. africanum *were commonly described that lay along the phenotypic continuum between *M. tuberculosis *and *M. bovis *[17]. We now understand that most strains formerly designated as *M. africanum *subtype II strains were actually *M. tuberculosis *[1,2,7,18-23], while strains formerly characterized as *M. africanum *subtype I can be segregated into two distinct genealogical clades on the basis of multiple genome sequence-level differences [1,2,7,23]. Several names have been given to each of the subtype I lineages in order to distinguish them. In this report we refer to the subtype I groupings as *M. africanum *West African-1 and *M. africanum *West African-2 [24,25]. For reference, as first described by Mostowy *et al. *[23], strains of *M. africanum *West African-1 (also known as clade 1 [26]) uniquely possess the long sequence polymorphism (LSP) RD713, while *M. africanum *West African-2 (also known as clade 2 [26]) carries the defining LSPs RD701 and RD702. Huard *et al. *[7], recently confirmed the clade specificity of these RDs, identified and validated the first SNPs restricted to either *M. africanum *West African-1 or *M. africanum *West African-2, and placed several additional previously known and novel polymorphisms into a unified phylogenetic context vis à vis *M. africanum *West African-1 and *M. africanum *West African-2.

In the present study, we characterized the content of known phylogenetically relevant RDs and SNPs in a blinded, and *M. africanum*-enriched, set of MTC strains isolated from TB patients in Ghana. The results of this evaluation established the utility of several consistent RD and SNP markers for *M. africanum *identification and clade differentiation and allowed us to settle upon a focused approach for future evaluations. In addition, novel SNPs were identified and validated against a large and diverse collection of MTC species and found to be specific to either *M. africanum *West African-1 (*Rv1332*^523^) or *M. africanum *West African-2 (*nat*^751^), thereby further expanding the limited number of genetic markers that can be used to unambiguously differentiate the two *M. africanum *lineages.

(This study contributed to the fulfillment of the Master's degree requirements by S.E.G.V.)

## Methods

### MTC strains analyzed

A total of 175 unique isolates that represent all of the presently described members of the MTC were included in the analysis and were derived from three strain collections, maintained at different institutions. One set of strains (*n *= 47) came from the National Reference Center for Mycobacteria in Forschungszentrum, Borstel, Germany and was collected in 2001-2003 from patients with pulmonary TB in Ghana. This set of Ghana strains was provided in a non-random blinded fashion but was known to contain both *M. africanum *and *M. tuberculosis *(as controls). All strains were previously characterized using the GenoType MTBC^® ^assay, as per the manufacturer's instructions, and these results were provided subsequent to the derivation of species identity using RD markers. A complete listing of the Ghana collection isolates by strain number accompanies a recent article by Wirth *et al. *[24] (excepting all *M. bovis *from Ghana and the non-*M. bovis *strains 10514/01, 1473/02, and 5357/02) and was recently made available as part of the MIRU-VNTR*plus *database http://www.miru-vntrplus.org/MIRU/index.faces[27]. Another 124 isolates were of a well-described strain collection from the Weill Medical College of Cornell University, New York. The extensive molecular characterization of the Cornell collection, and a complete listing by MTC species, unique identifier, and origin, was previously reported [7]. Only one isolate from that collection (*M. tuberculosis *strain W) was not included in the current evaluation. This sampling was composed of "*M. canettii*" (*n *= 5), *M. tuberculosis *(*n *= 44), *M. africanum *West African-1 (*n *= 12) (note: given previously as *M. africanum *subtype Ib), *M. africanum *West African-2 (*n *= 18) (note: given previously as *M. africanum *subtype Ia), the dassie bacillus (*n *= 4), the oryx bacillus (*n *= 2), *M. microti *(*n *= 10), *M. pinnipedii *(*n *= 7), *M. caprae *(*n *= 1), *M. bovis *(*n *= 14), and *M. bovis *BCG (*n *= 8). Lastly, 15 DNA samples were provided from the collection of the National Institute for Public Health and the Environment (RIVM), Bilthoven, the Netherlands [28]. These included strains of *M. tuberculosis *(strains 13 and 22), "*M. canettii*" (strains 116 and 119), *M. africanum *West African-1 (strain 92), *M. africanum *West African-2 (strains 6 and 85), *M. microti *(strains 25 and 62), *M. pinnipedii *(stains 76 and 81), *M. bovis *(117 and 128), and *M. bovis *BCG (2 and 71) (note: some strain identities are corrected as per [2,22]). The 4 strains underlined in the above were unique and the remaining 11 were also included in the Cornell collection [7]. All strains from the Ghana collection were screened for every marker of interest while strains of the Cornell and RIVM collections were screened selectively, as described in each respective section of the Results.

#### PGG Analysis

Frequently observed SNPs in the genes *katG*^463 ^and *gyrA*^95 ^are routinely assessed in order to broadly categorize isolates into defined MTC phylogenies, known as principal genetic groups (PGG) [29]. The distribution of SNPs in *katG*^463 ^and *gyrA*^95 ^suggests that PGG1 *M. tuberculosis *strains more closely resemble the most recent common ancestor of all *M. tuberculosis *strains than PGG2 strains, and PPG2 strains more so than PGG3 strains. MTC species along the *M. africanum*→*M. bovis *evolutionary track are also PGG1 [1,2]. SNP analysis of *katG*^203 ^was used to further segregate PGG1a isolates from PGG1b strains [7,30]. Representatives of each PGG were included in the Cornell collection of MTC strains.

### MTC PCR-typing Panel

In previous reports we described [2], and then expanded upon [7], a PCR-based protocol for the differentiation of the various MTC species on the basis of genomic deletions. This MTC PCR-typing panel targets eight independent loci for amplification (*16S rRNA*, *cfp32 *[*Rv0577*], MiD3 [IS*1561'*], RD4 [*Rv1510*], RD7 [*Rv1970*], RD1 [*Rv3877-Rv3878*], RD9 [*Rv2073c*], and RD12 [*Rv3120*]), each of which either results in an amplicon of an expected size or fails, depending upon the genomic content of the MTC strain being evaluated. The resulting band-pattern that is observed following agarose gel electrophoresis is indicative of MTC species identity. Of note, the RD12 target region in *M. bovis *and *M. caprae *overlaps a specific LSP in "*M. canettii*" (RD12^can^), while the RD1 target region in *M. bovis *BCG overlaps a specific LSP in the dassie bacillus (RD1^das^). With this protocol, the pattern of bands for *M. microti *and *M. pinnipedii *are identical, while the pattern of bands for the orxy bacillus is the same as that of *M. africanum *West African-2. The MTC PCR-typing panel has been successfully applied to collections of MTC strains from Rio de Janeiro, Brazil, and Kampala, Uganda, in order to characterize the diversity of MTC species within these locales [21,31].

### PCR amplification primers and conditions

Purified DNA was prepared for PCR as previously described [2]. For some strains, culture thermolysates (80°C for 30 min) were used as the source of DNA in PCR amplifications. The primers used for the MTC PCR-typing panel, the RD^Rio ^flank multiplex, RD174, RD701, RD702, RD711, RD713, in addition to targets containing the the *pks*15/1 micro-deletions and SNPs at *aroA*^285^, 3'*cfp32*^311^, *gyrA*^95^, *gyrB*^1450^, *hsp65*^540^, *katG*^203^, *katG*^463^, *PPE55*^2148^, *PPE55*^2154^, *narGHJI *^-251^, RD13^174^, *rpoB*^1049^, *rpoB*^1163^, *Rv1510*^1129^, and TbD1^197^, were the same as described earlier [7,32,33]. For analysis of the loci RD8, RD9, RD10, RD701, and TbD1 additional new site-specific 3-primer combinations were designed for each, similar to as previously detailed [32], and each included two deletion flanking primers and one primer internal to the deletion. The 3-primer PCRs were each designed to amplify a product of one size when the target locus is intact or to produce a different band size when a known LSP is present. New primers were also designed to amplify a 1069-bp *nat *gene fragment and the SNP-containing targets in *nat*^751 ^and *Rv1332*^523^. New primers, along with expected band sizes and the PCR program used to amplify, are listed in Table 1. The general PCR protocol was identical to that used previously [2,7]. PCR amplification from purified DNA was performed using the following cycling conditions: Program 1a (with an initial denaturation step of 5 min at 94°C, followed by 45 cycles of 1 min at 94°C, 1 min at 60°C, and 1 min at 72°C, and ending with a final elongation step for 10 min at 72°C) or program 2a (similar to program 1a but with an annealing temperature of 65°C). PCR testing of DNA thermolysates was performed in a similar manner using the following cycling conditions: Program 1b (with an initial denaturation step of 5 min at 94°C, followed by 45 cycles of 1 min at 94°C, 1 min at 60°C, and 4 min at 72°C, and ending with a final elongation step for 10 min at 72°C) or program 2b (similar to program 1b but with an annealing temperature of 65°C). Programs 1b and 2b were also used to amplify from purified DNA when potential target PCR fragments were greater than 1,250 bp. PCR products were visualized as previously described by agarose gel electrophoresis [2]. Negative or unexpected positive PCR results were repeated at least once for confirmation. Importantly, all PCR tests included parallel samples containing DNA of *M. tuberculosis *strain H37Rv (ATCC 27294^T^) and either *M. africanum *West African-1 strain Percy16, *M. africanum *West African-2 strain ATCC 25420^T^, or *M. bovis *strain ATCC 19210^T^, where appropriate, as controls. All controls consistently provided the expected results for each particular marker screened. Negative control PCRs, lacking input DNA, were also included to control for DNA contamination.

**Table 1:** New primers used in this study.

Target Locus	Primer Name	Nucleotide Sequence	PCR Program	Size (bp)^1^
New 3-primer combinations				
RD8	RD8flnkF	5' CAT GCT AAG CAG ATC GTC AGT TTT GA 3'	1a, 1b	289/485
	RD8iF	5' GCC GCA TTG TCG GGG TGC GAT TCC CAC ACC 3'		
	RD8flnkR	5' CGG TTC CGG CGG GCT CCG GAT TGC TGT ACT 3'		
RD9	RD9flnkF	5' ACT CCC AGC GCT CGG CGG TGA CGG TAT CGT 3'	1a, 1b	293/499
	RD9iR	5' ATT CCG TGG GCG CTG CGG CCA ATG TTT GTT 3'		
	RD9flnkR	5' GTG GCT CGG CAC GCA CAA CTC GTT CAA CAG 3'		
RD10	RD10flnkF	5' GCG CCA CCT CGG CCG GAT TCC TGC AAC CAT 3'	1a, 1b	291/478
	RD10iR	5' TTC GGC CTT GCC GTC ATA GCG CAA TAG CGA 3'		
	RD10flnkR	5' CTC GGC GGC AAG TCG GCG GCC ATC ATT CTC 3'		
RD701	RD701flnkF	5' ACT CGC CGG CTG TGC AGG TGG TCG TT 3'	1a, 1b	350/487
	RD701iR	5' CCA AAA TTG TCG CCC TTC AGT GCG GTA TCC 3'		
	RD701flnkR	5' GAG GGG CAG CGC GGG GAA GTC G 3'		
TbD1	TbD1flnkF	5' CTA CCT CAT CTT CCG GTC CA 3'	1a, 1b	298/485
	TbD1iF	5' AAG GAA CTG CGA GAT AGG ATC GCC AAT TTC 3'		
	TbD1flnkR	5' CAT AGA TCC CGG ACA TGG TG 3'		
New primers for PCR-RFA				
*Rv1332*^523^	Rv1332F	5' GCC CTG CGC AGC CTG CAC GAA CCT GAG ATT 3'	1a, 1b	344
	Rv1332R	5' GGA TGC CCC CGA CGT CGG TGA TGG AGT TCA 3'		
*nat*^751^	nat751F	5' ACC CGG CAT CGA AGT TCG TCA CGG GAC TGA 3'	2a, 2b	766
	nat751R	5' TGG TGT ACC AGG GGG CAC CGC AAA CCA G 3'		
New amplification primers				
*nat*	natF	5' ATC GGT GCG ACA TAG TTG G 3'	2a, 2b	1069
	natR	5' GCC TTC TGC TCA AAG TTG CT 3'		
Additional sequencing primers				
*nat*	natiF	5' CAC CGA CCT CAC CGC TTC 3'		
	natiR	5' GTC CTC GAG CCG ATA AGG TT 3'		
Corrected primer from ref. 7				
*katG*^203^	katG203R	5' CAA GAA GCT CTC ATG GGC GGA CCT GAT TGT 3'		

^1 ^for RD loci, expected band sizes given as intact/deletion present

PCR of the *nat *gene (1069 bp) was performed by a slightly different protocol. PCR Program 2a and a PCR reaction mix in 50 μl, with 40 pmol of each primer, 5 mM MgCl_2_, 0.2 mM dNTPs, 1U *Taq *polymerase (Invitrogen, Brazil), PCR-buffer (10 mM Tris-HCl, 1.5 mM MgCl_2_, 50 mM KCl, pH 8.3) (Invitrogen, Brazil), 10% glycerol, and 10 ng of target DNA were used in this case.

It should be noted that the *M. africanum *West African-1- and *M. africanum *West African-2-restricted LSPs were amplified by RD flanking primers [23] and analyzed as previously described [7] with the results based upon a size estimation of the PCR products on agarose gel. PCR amplification of RD713 in *M. africanum *clade 1 strains typically yields a 2,798 bp amplicon, while amplification of this locus in other MTC strains either results in a 4,248 bp product (PGG2 and PGG3 *M. tuberculosis*) or no PCR product (PGG1a MTC species with the partially overlapping RD7 deletion and PGG1b *M. tuberculosis *which possess additional genomic content at this locus [7]). In PCR amplification of RD711, most, but not all, *M. africanum *clade 1 strains are expected to yield a 944 bp amplicon while the remaining *M. africanum *West African-1 strains and MTC species amplify a 2,885 bp product. With respect to RD701, all *M. africanum *West African-2 strains are expected to generate a 340 bp amplicon while strains from the other MTC species amplify a 2,081 bp PCR fragment. Likewise, for RD702, all *M. africanum *West African-2 strains are expected to amplify a 732 bp product while strains from the other MTC species produce a 2,101 bp PCR fragment. For this study, RD9 (as part of the MTC PCR typing panel), TbD1, and RD701 were evaluated by both 2-primer and 3-primer PCR tests.

### SNP analysis by PCR-RFA and sequencing

Characterization of the SNPs at *gyrA*^95^, *gyrB*^1450^, *hsp65*^540^, *katG*^203^, *katG*^463^, *narGHJI *^-251^, *rpoB*^1049^, *rpoB*^1163^, and *Rv1510*^1129 ^was performed by PCR-RFA [7,33]. For the analysis of the SNPs in *aroA*^177^, 3'*cfp32*^311^, *mmpL6*^551^, *nat*^751^, and TbD1^197 ^novel PCR-RFA procedures were developed, similar to those previously detailed [2,7]. The restriction enzymes and expected digest band sizes for each PCR-RFA are listed in Table 2. Amplified products from *M. tuberculosis *H37Rv and a second appropriate MTC species (see above) were included in all digest reactions as controls. All unexpected digestion results were repeated least once for confirmation. For each PCR-RFA evaluation, the PCR fragments from at least one strain of each digest pattern were sequenced in order to confirm the presence or absence of the target SNP.

**Table 2:** Summary of PCR-RFA protocols used in this study^1^

Locus	Restriction enzyme	MTC species	Predicted digest pattern (bp)
*katG*^463^	*Bst*NI	PGG1 MTC	12, 59, 106, 174
		PGG2, PGG3 *M. tuberculosis*	12, 59, 280
			
*gyrA*^95^	*Ale*I	PGG1, PGG2 MTC	161, 193
		PGG3 *M. tuberculosis*	354
			
*narGHJI *^-251^	*Sau*3AI	"modern" *M. tuberculosis*	155
		Remaining MTC	69, 86
			
*gyrB*^1450^	*Taq*1α	*"M. canettii", M. tuberculosis*	6, 21, 74, 96, 129, 270, 444
		Remaining MTC	6, 21, 74, 96, 129, 163, 107, 444
			
*katG*^203^	*Bst*NI	*"M. canettii", M. tuberculosis*, *M. africanum *WA-1	140, 230
		Remaining MTC (PGG1a)	370
			
*3'cfp32*^311^	*Bst*NI	*M. tuberculosis*, *M. africanum *WA-1	28, 34, 75, 235^2^
		Remaining MTC (PGG1a)	34, 75, 263
			
*aroA*^117^	*Bss*HII	*M. africanum *WA-1	177, 254
		Remaining MTC	104, 150, 177
			
TbD1^197^	*Tse*I	*M. africanum *WA-1	46, 454
		Remaining MTC	46, 193, 261^3^
			
*Rv1510*^1129^	*Nru*I	*M. africanum *WA-2, dassie bacillus	192, 841
		Remaining MTC	1033^4^
			
*hsp65*^540^	*Afl*III	*M. africanum *WA-2	49, 392
		Remaining MTC	441
			
*nat*^751^	*Bcg*I	*M. africanum *WA-2	111, 177, 208, 270
		Remaining MTC	111, 208, 447
			
*rpoB*^1049^	*Sau*3AI	*M. africanum *WA-2	11, 12, 18, 69, 87, 163
		Remaining MTC, *M. africanum *WA-2	11, 12, 18, 69, 250
			
*rpoB*^1163^	*Bst*UI	*M. africanum *clade 2	28, 332
		Remaining MTC, *M. africanum *WA-2	28, 79, 253
			
*mmpL6*^551^	*Hpy*166III	*"M. canettii", M. tuberculosis*, *M. africanum *WA-1, *M. africanum *WA-2, dassie bacillus	155, 298^3^
		Remaining MTC	453

WA, West African

^1 ^This table contains the correction of errors from ref. [7]

^2 ^"*M. canettii*" fails to PCR amplify

^3 ^'modern' *M. tuberculosis *fails to PCR amplify

^4 ^*M. bovis *fails to PCR amplify

Because it was not possible to develop a PCR-RFA based approach for characterization of the SNPs at *PPE55*^2148^, *PPE55*^2154^, RD13^174^, and *Rv1332*^523^, SNP analysis for these markers was performed by direct sequencing of the PCR products. The same procedure was used for verification of micro-deletions in the *pks15/1 *locus [34]. In most cases, the primers for PCR amplification primers were also used for sequencing, as previously described [2,7], with the exception of the 1069 bp *nat *fragment which was also sequenced using internal primers Table 1). Sequencing was performed using the BigDye Terminator kit (PE Applied Biosystems) on an ABI 3730 DNA Analyzer, either at the Cornell University BioResource Center (Ithaca, NY) http://www.brc.cornell.edu or at the Oswaldo Cruz Foundation (PDTIS DNA Sequencing Platform/FIOCRUZ, Rio de janeiro, RJ.); http://www.dbbm.fiocruz.br/PDTIS_Genomica/) and the results were analysed as previously described [2,7].

### Nucleotide sequence accession numbers

Gene fragment sequences containing novel SNPs were submitted to GenBank for *M. africanum *West African-1 (*Rv1332*^523^; accession number FJ617580) and *M. africanum *West African-2 (*nat*^751^; accession number FJ617579). Previously identified polymorphic gene fragment sequences are now available for *M. africanum *West African-1 (*aroA*^285 ^[FJ617581] and TbD1^197 ^[FJ617582]) and *M. africanum *West African-2 (*hsp65*^540 ^[FJ617583]; *Rv1510*^1129 ^[GU270931]; and *rpoB *variants [FJ617584, FJ617585, FJ617586]).

## Results

### Genetic characterization of MTC isolates by PCR deletion analysis

**Figure 1:**
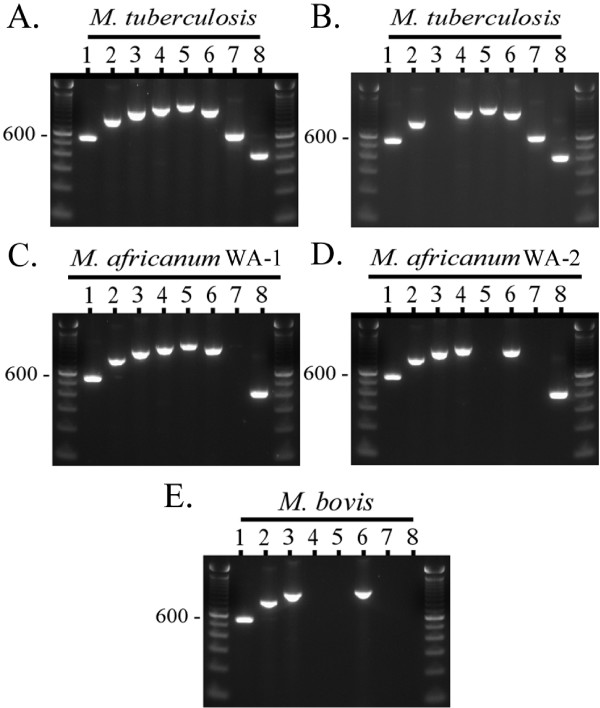
**The composite MTC PCR typing panel**. Illustrated is the MTC PCR typing panel output pattern for **A**) a typical *M. tuberculosis *strain, **B**) a secondary pattern seen in some Cameroon genotype *M. tuberculosis *strains from Ghana, **C**) a typical *M. africanum *West African-1 strain, **D**) a typical *M. africanum *West African-2 strain, and **E**) a typical *M. bovis *strain [7]. PCR products and the 100-bp ladder (unlabelled lanes) were visualized by agarose gel electrophoresis and ethidium bromide staining. Lanes: 1, *16S rRNA*; 2, *cfp32 *(*Rv0577*); 3, MiD3 (IS*1561'*); 4, RD4 (*Rv1510*); 5, RD7 (*Rv1970*); 6, RD1 (*Rv3877-Rv3878*); 7, RD9 (*Rv2073c*); 8, RD12 (*Rv3120*). WA - West African.

**Figure 2:**
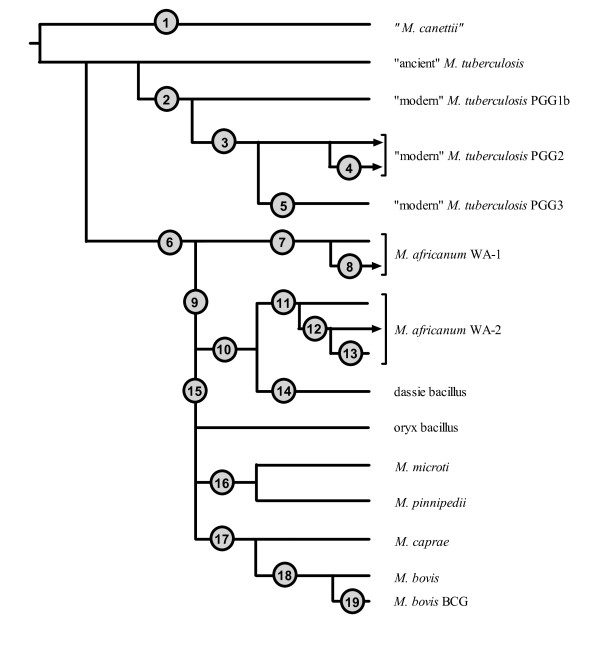
**Summary diagram and phylogenetic postitions of the genomic markers interrogated against the Ghana MTC strain collection**. Shown are the various major divisions of the MTC segregated according to the presence or absence of inter-species-, intra-species-, and sublineage-specific polymorphisms. Circles are placed at points in evolutionary history beyond which each strain that was evaluated possessed a consistent set of polymorphisms. The nodes are numbered in the figure as follows to denote: 1. RD12^can^, 3' *cfp32 *deletion; 2. TbD1, *narGHJI *^-215^; 3. *pks15/1 *(7-bp deletion), *katG*^463^; 4. undefined deletion at the RD^Rio^/MiD3 locus; 5. *gyrA*^95^; 6. RD9, *gyrB*^1450^; 7. RD713, TbD1^197^, *aroA*^285^, *Rv1332*^523^; 8. RD711; 9. RD7, RD8, RD10, *pks15/1 *(6-bp deletion), *katG*^203^, 3'*cfp32*^311^, RD13^174^, *PPE55*^2148^, *PPE55*^2154^; 10. *Rv1510*^1129^; 11. RD701, RD702, *hsp65*^540^, *nat*^751^; 12. *rpoB*^1163^; 13. *rpoB*^1049^; 14. RD1^das^; 15. *mmpL6*^551^; 16. MiD3; 17. RD12, RD13; 18. RD4; 19. RD1^BCG^. Lineages that include strains from the Ghana collection are terminated with arrowheads. Note that distances are arbitrary and do not reflect the number of phylogenetically relevant polymorphisms present at each juncture. TbD1-positive *M. tuberculosis *is also known as "ancient" *M. tuberculosis *and TbD1-negative *M. tuberculosis *is also known as "modern" *M. tuberculosis *[1]. WA - West African.

For this study we applied the MTC PCR-typing panel to a blinded, *M. africanum*-enriched, challenge collection of MTC strains isolated from patients with TB in Ghana (*n *= 47). As a result, 18 *M. tuberculosis *isolates, 20 strains of *M. africanum *West African-1, and 9 *M. africanum *West African-2 strains were putatively differentiated [7]. Strains were identified as *M. tuberculosis *by the successful amplification of targets internal to the RD9 and RD12/RD12^can ^loci. Strains were identified as *M. africanum *West African-1 on the basis of failure of amplification of the RD9 locus but the successful amplification of the RD7 target region, while *M. africanum *West African-2 strains were putatively identified on the basis of failure of amplification of the RD9 and RD7 loci but the successful amplification of regions within the RD1^bcg^/RD1^das^, RD4, and RD12 loci. No *M. bovis *strains (which would have shown a pattern lacking in amplicons for RD4, RD7, RD9, and RD12) or other MTC species were identified (see ref. 7 for the expected MTC PCR typing panel patterns of "*M. canettii*", *M. microti*, *M. pinnipedii*, and the dassie bacillus). Of note, all strains amplified for the *cfp32 *(*Rv0577*) gene, a target that has been previously proposed to be MTC-restricted and may be necessary for pathogenesis [2,7,35]. The segregation of *M. tuberculosis *from *M. africanum *in this collection by the MTC PCR typing panel paralleled the results derived from the GenoType MTBC^® ^assay, which assigned these isolates as either *M. tuberculosis *(*n *= 18) or *M. africanum *subtype I (*n *= 29). These identifications were consistent with independently derived data for this strain set [24]. Fig. 1 illustrates a typical MTC PCR-typing panel profile for *M. tuberculosis*, *M. africanum *West African-1, *M. africanum *West African-2, and *M. bovis*. A summary of all molecular test results derived in this study is provided in Table 3 and illustrated schematically in Fig. 2. With respect to the RD markers interrogated above, note their phylogenetic positions in Fig. 2 at nodes 1, 6, 9, 14, and 16-19.

**Table 3:** Summary of results targeting MTC polymorphic loci

Target Locus	Node #(s) in Fig. 2	*M. tuberculosis *(*n*)	*M. africanum *WA-1 (*n*)	*M. africanum *WA-2 (*n*)	Other MTC (*n*)
RD loci					
RD12^can^/RD12 ^1^	1, 17	intact (18)	intact (20)	intact (9)	
3'*cfp32*	1	intact (18)	intact (20)	intact (9)	
TbD1	2	deleted (18)	intact (20)	intact (9)	
*pks15/1*	3, 9	7-bp deletion (18)	intact (20)	6-bp deletion (9)	
*PPE55 *^2^	4, 16	intact (9) fail (9)	intact (20)	intact (9)	
MiD3/RD^Rio 2 ^(IS*1561' *+ *PPE55*)	4, 16	intact (9) fail (9)	intact (20)	intact (9)	
RD9	6	intact (18)	deleted (20)	deleted (9)	
RD713 ^3^	7	fail (18)	deleted (20)	fail (9)	
RD711	8	intact (18)	deleted (20)	intact (9)	
RD7 ^3^	9	intact (18)	intact (20)	deleted (9)	
RD8	9	intact (18)	intact (20)	deleted (9)	
RD10	9	intact (18)	intact (20)	deleted (9)	
RD701	11	intact (18)	intact (20)	deleted (9)	
RD702	11	intact (18)	intact (20)	deleted (9)	
RD1^das^/RD1^bcg 4^	14, 19	intact (18)	intact (20)	intact (9)	
RD13	17	intact (18)	intact (20)	intact (9)	
RD4	18	intact (18)	intact (20)	intact (9)	
SNP loci					
*narGHJI *^-215^	2	C→T (18)	no Δ (20)	no Δ (9)	
*katG*^463^	3	CTG→CGG (18)	no Δ (20)	no Δ (9)	
*gyrA*^95^	5	no Δ (18)	no Δ (20)	no Δ (9)	
*gyrB*^1450^	6	no Δ (18)	G→T (20)	G→T (9)	
TbD1^197^	7	fail (18)	C→T (20)	no Δ (9)	
*aroA*^285^	7	no Δ (18)	G→A (20)	no Δ (9)	
*Rv1332*^523^	7	no Δ (27)	G→T (32)	no Δ (11)	no Δ (15)
*katG*^203^	9	no Δ (18)	no Δ (20)	ACC→ACT (9)	
3'*cfp32*^311^	9	no Δ (18)	no Δ (20)	G→A (9)	
RD13^174^	9	no Δ (18)	no Δ (20)	G→A (9)	
*PPE55*^2148^	9	no Δ (9) fail (9)	no Δ (20)	A→G (9)	
*PPE55*^2154^	9	no Δ (9) fail (9)	no Δ (20)	A→G (9)	
*Rv1510*^1129^	10	no Δ (18)	no Δ (20)	G→A (9)	
*hsp65*^540^	11	no Δ (18)	no Δ (20)	C→G (9)	
*nat*^751^	11	no Δ (63)	no Δ (32)	G→A (27)	no Δ (53)
*rpoB*^1163^	12	no Δ (18)	no Δ (20)	C→T (9)	
*rpoB*^1049^	13	no Δ (18)	no Δ (20)	no Δ (9)	
*mmpL6*^551^	15	fail (18)	no Δ (20)	no Δ (9)	
Other targets					
*cfp32*		intact (18)	intact (20)	intact (9)	

^1,2,3,4 ^pairs of partially overlapping LSPs

WA, West African; Fail, no PCR amplification; bp, base pair; Δ, change

An exception to the common *M. tuberculosis *MTC PCR-typing panel profile occurred with 9 *M. tuberculosis *strains from Ghana, which failed to amplify the IS*1561' *target (see Fig. 1B). Previously, strains with this particular band pattern were found to share a clonal deletion called RD^Rio ^that defines a major, newly recognized, lineage of *M. tuberculosis *that is the predominant cause of TB in Rio de Janeiro, Brazil, and that has disseminated to many countries around the world [7,31,32]. However, multiplex PCRs for both the RD^Rio ^LSP and the coincident RD174 deletion [32] showed that these Ghanaian strains were not RD^Rio ^genotype *M. tuberculosis*. Rather, data from the MIRU-VNTR*plus *website identified these strains as being of the RD726-harboring Cameroon genotype (ST61 and variants) and lists the strains as lacking IS*1561' *[27]. The Cameroon genotype therefore appears to possess an undefined LSP of IS*1561' *that overlaps RD^Rio ^(Fig. 2; see node 4) and the MiD3 locus in *M. microti *and *M. pinnipedii *(Fig. 2; see node 16) [7,31].

In addition to the MTC PCR-typing panel, some PCR targets used in SNP analysis, as will be described below, amplify from genomic regions that are deleted is some MTC species or lineages [7]. The successful amplification of the 3'*cfp32 *and RD13 loci in all the strains of the Ghana collection confirmed the species distribution obtained using the MTC PCR-typing panel, as these targets are deleted in either "*M. canettii*" (Fig. 2; see node 1) or both *M. caprae *and *M. bovis *(Fig. 2; see node 17), respectively [7]. Furthermore, *PPE55 *is located proximal to IS*1561' *and so the failure to amplify *PPE55 *from the 9 Cameroon genotype *M. tuberculosis *isolates is consistent with a single genomic deletion in the region of IS*1561' *(Fig. 2; see node 4). Lastly, TbD1 is an important phylogenetic marker that categorically divides *M. tuberculosis *into two major lineages [1]. All *M. tuberculosis *isolates in the Ghana collection failed to amplify from targets internal to TbD1 (Fig. 2; see node 2), while all *M. africanum *clades 1 and 2 strains yielded an amplicon of the correct size, consistent with the previous finding that isolates from the *M. africanum*→*M. bovis *evolutionary tract are all TbD1-positive and likely a branch off of a TbD1-positive *M. tuberculosis *lineage [1,24].

We next evaluated the Ghana strain collection by PCR (using LSP flanking primers) for RDs that have been described previously as being either specific to *M. africanum *West African-1 (RD713), restricted to a subgroup of *M. africanum *West African-1 (RD711), or specific to *M. africanum *West African-2 (RD701 and RD702) [7,23]. All *M. africanum *West African-1 strains (*n *= 20) yielded amplification products for RD711 and RD713 of shorter band sizes that were consistent with amplicons that bridge a deletion (Fig. 2; see nodes 7 and 8). All *M. tuberculosis *strains (*n *= 18) contained the RD711 and RD713 regions, while each *M. africanum *West African-2 strain (*n *= 9) yielded PCR fragments suggestive of intact RD711. Each *M. africanum *West African-2 strain (*n *= 9) also failed to produce any amplification products from the RD713 locus region, as expected, owing to the overlapping RD7 [7]. Likewise, all *M. africanum *West African-2 strains produced shortened RD701 and RD702 amplicons (Fig. 2; see node 11), while each *M. tuberculosis *and *M. africanum *West African-1 strain exhibited PCR fragments representative of intact sequences within these loci. The *M. africanum *clade-specific bridge-deletion PCR results were therefore congruent with the MTC PCR-typing panel data.

A drawback, however, of the MTC PCR-typing assay as it was designed is that overlapping polymorphisms may occur in the target regions of the panel. Such hypothetical LSPs would therefore have the potential to cause a failure in amplification and to confuse the interpretation of banding patterns which may, in turn, lead to erroneous species determinations. To begin to address this issue, with respect to loci relevant to the species within the current Ghana collection, we developed new 3-primer combination sets for RD8, RD9, RD10, RD701, and TbD1 (Table 1). As was expected from previous phylogenetic evaluations [1,3,7], each of the test loci were found to be intact in the Ghana collection PGG2 *M. tuberculosis *strains, excepting TbD1. Moreover, excepting RD9, each of the studied RDs were intact in the *M. africanum *West African-1 strains, while in the *M. africanum *West African-2 strains only TbD1 remained intact, i.e. the RDs 8-10 and RD701 were deleted. Overall, no inconsistencies were observed with respect to species identification within the Ghana MTC strain collection across the different strategies for PCR deletion analysis that were employed.

### Genetic characterization of MTC isolates by SNP analysis

For the second stage of this study we screened the Ghana MTC collection for known phylogenetically relevant SNPs. With respect to the *M. tuberculosis *strains, we determined that all were PGG2 (*n *= 19) (Fig. 2; see nodes 3 and 5). Consistent with this determination, the 7-bp *pks15/1 *micro-deletion was observed in all the *M. tuberculosis *strains; this polymorphism is positioned at the same point along the MTC evolutionary tree as the *katG*^463 ^CTG→CGG SNP that marks PGG2 *M. tuberculosis *strains (Fig. 2; see node 3). Likewise, an SNP in the *narGHJI *operon promoter (-215 C→T), that is phylogenetically coincident with TbD1 [33] was also present in all of the Ghanaian *M. tuberculosis *isolates evaluated (Fig. 2; see node 2). Lastly, the *gyrB*^1450 ^G→T polymorphism (also a target of the GenoType MTBC^® ^assay [14-16]) is known to coincide with the RD9 deletion and likewise segregated the *M. tuberculosis *isolates from the strains of the *M. africanum *strains (Fig. 2; see node 6).

The following considers SNPs that inform the phylogenetic interrelationships among most of the non-*M. tuberculosis *MTC species. First, all the *M. africanum *strains (*n *= 28) were PGG1. Previously, an ACC→ACT SNP at *katG*^203 ^has been used to segregate PGG1 strains into PGG1a and PGG1b [30]. Huard *et al. *[7] reported that this SNP is present in *M. africanum *West African-2 and all downstream species in the MTC evolutionary tree (Fig. 2; see node 9). As expected, the Ghana collection *M. africanum *West African-1 strains were determined to be PGG1b, while the *M. africanum *West African-2 strains were PGG1a by *katG*^203 ^analysis. Additional inter-species-specific SNPs that colocalize with the *katG*^203 ^SNP and segregate the *M. africanum *clades (and are also notably coincident with RD7, RD8, and RD10) have also been reported at 3'*cfp32*^311 ^(G→A), *PPE55*^2148 ^(A→G), *PPE55*^2154 ^(A→G), and RD13^174 ^(G→A), in addition to a 6-bp *pks15/1 *micro-deletion (Fig. 2; see node 9) [7,34]. These loci were interrogated and indeed found to partition the *M. africanum *West African-2 strains from the *M. africanum *West African-1 and *M. tuberculosis *strains of the Ghana collection, consistent with previous reports [7,34]. Lastly, we also screened for an inter-species-specific SNP in *mmpL6*^551 ^(AAC→AAG) [1,7] that is not observed in *M. africanum *West African-1, *M. africanum *West African-2, nor the dassie bacillus, but is present in all of the remaining distal species along the oryx bacillus→*M. bovis *evolutionary track of the MTC phylogenetic tree [1,7,26]. As was expected, we found *mmpL6*^551 ^to be unaltered in the *M. africanum *West African-1 and West African-2 strains of the Ghana MTC collection (Fig. 2; see node 15). The *mmpL6*^551 ^SNP occurs within a TbD1 locus gene and was thus deleted in the TbD1-negative *M. tuberculosis *strains of the Ghana collection.

We then investigated SNPs that have been previously described to be restricted to either *M. africanum *West African-1 or *M. africanum *West African-2 within the MTC [7]. SNPs at *aroA*^285 ^(G→A) and TbD1^197 ^(C→T) were found to be limited to the *M. africanum *West African-1 strains of the Ghana MTC collection, thereby coinciding with the *M. africanum *West African-1-specific LSP RD713 (Fig. 2; see node 7). Point mutations at *Rv1510*^1129 ^(G→A), *hsp65*^540 ^(C→G), and *rpoB*^1163 ^(C→T) were also screened and found to be restricted to the *M. africanum *West African-2 strains (Fig. 2; see nodes 10-12); a previously noted sublineage-specific SNP at *rpoB*^1049 ^(C→T) was not observed (Fig. 2; see node 13). However, from previous data [7], only *hsp65*^540 ^has been shown to be truly *M. africanum *West African-2-specific and to associate pylogenetically with RD701 and RD702. In fact, *Rv1510*^1129 ^was previously found to be an inter-species-specific SNP that *M. africanum *West African-2 shares with the dassie bacillus, and is indicative of a common ancestor between these species, while not all *M. africanum *West African-2 strains possess the *rpoB*^1163 ^and *rpoB*^1049 ^SNPs [7]. These latter point mutations appear to have been acquired in a step-wise sequential order and to define the branch points of sublineages within the *M. africanum *West African-2 species. All Ghana *M. africanum *West African-2 strains evaluated in this study therefore fell into the second of three potential *rpoB *sequence-based sublineage branches. Overall, each of the known MTC inter-species-specific, species-specific, and sublineage-specific SNPs for which the Ghana MTC collection was evaluated were entirely consistent with the current RD analyses and showed a species distribution that paralleled previous descriptions [7].

### Identification of a novel Mycobacterium africanum West African-1-specific Rv1332^523 ^SNP

In the process of sequencing the RD711 bridge amplicon to confirm its correct amplification in an *M. africanum *West African-1 strain, we noted a nonsynonomous G→T SNP in the region 5' of the RD711 deletion breakpoint and within the *Rv1332 *gene, affecting nucleotide 523 (*Rv1332*^523^; V175L). To investigate the distribution of this *Rv1332*^523 ^SNP amongst the MTC species, we generated a new primer pair to amplify the SNP-containing region upstream of RD711. We then performed PCR and sequence analysis of the amplified products upon samples from select MTC strains of the Cornell collection representing each of the MTC species and major *M. tuberculosis *lineages, i.e., "*M. canettii*" (*n *= 2), TbD1-positive *M. tuberculosis *PGG1 (*n *= 2), TbD1-negative *M. tuberculosis *PGG1 (*n *= 2), *M. tuberculosis *PGG2 (*n *= 2), *M. tuberculosis *PGG3 (*n *= 3), *M. africanum *West African-1 (*n *= 12), *M. africanum *West African-2 (*n *= 2), the dassie bacillus (*n *= 2), the oryx bacillus (*n *= 2), *M. microti *(*n *= 2), *M. pinnipedii *(*n *= 2), *M. caprae *(*n *= 1), *M. bovis *(*n *= 2), and *M. bovis *BCG (*n *= 2). Only the 12 *M. africanum *West African-1 strains possessed the *Rv1332*^523 ^substitution. When the Ghana collection was subsequently evaluated (*n *= 47), the *Rv1332*^523 ^SNP was likewise restricted to the 20 *M. africanum *West African-1 strains. In total, 85 MTC isolates were screened, 32 of which were *M. africanum *West African-1. The data thus supported that the *Rv1332*^523 ^SNP is a specific marker for *M. africanum *West African-1 and is only the third such polymorphism reported to date (Fig. 2; see node 7) [7].

### Identification of a novel Mycobacterium africanum West African-2-specific nat^751 ^SNP

Previously, the *nat *(*Rv3566c*) gene product arylamine *N*-acetyltransferase has been investigated as a potential contributor to reduced isoniazid susceptibility in *M. tuberculosis *[36]. In the course of those investigations, SNPs were identified in the *nat *gene that were restricted to different *M. tuberculosis *lineages. We found a novel nonsynonomous G→A SNP in two *M. africanum *West African-2 strains at *nat *nucleotide 751 (*nat*^751^; E251K) upon amplification and sequencing of a 1069-bp *nat *fragment using samples from a subset of MTC representative strains (RIVM collection; *n *= 15). Test sequencing of the 1069-bp *nat *amplicon from 16 MTC strains from the Cornell collection supported the limited distribution of the *nat*^751 ^SNP. We then developed a PCR-RFA protocol for the *nat*^751 ^SNP, amplifying a shorter product using new primers and employing the restriction enzyme *Bcg*I, and applied the protocol to all strains of both the Cornell (*n *= 124) and Ghana collections (*n *= 47). Consistent with the preliminary test results, all MTC isolates amplified *nat *successfully. However, only the 27 *M. africanum *West African-2 strains possessed the *nat*^751 ^polymorphism, as determined by PCR-RFA. The West African-2 strains showed a 4-band digest pattern on agarose gel electrophoresis as opposed to the remaining MTC strains that showed a 3-band digest pattern (see Table 2). Thus, this SNP appears to be a specific marker for *M. africanum *West African-2 (*n *= 175 unique MTC strains evaluated in total) and is only the second SNP reported to be restricted to this clade (Fig. 2; see node 11) [7]. Of note, both the *nat*^751 ^and *hsp65*^540 ^*M. africanum *West African-2-specific SNPs are present in the genomic sequencing project of *M. africanum *strain GM041182 that is currently nearing assembly completion http://www.sanger.ac.uk/sequencing/Mycobacterium/africanum/.

## Discussion

*M. africanum *has been reported to be an important cause of TB in the West African countries of Guinea-Bissau (52%) [37], The Gambia (38%) [38], Sierra Leone (24%) [39], Senegal (20%) [17], Burkina Faso (18.4%) [40], Cameroon (9%) [41], Nigeria (8%) [42], and Côte D'Ivoire (5% of cases) [22]. *M. africanum *has also been identified in the West African countries of Benin, Mauritania, and Niger [7,43]. Many of the previous *M. africanum *reports appeared, however, before molecular markers distinguished two different clades within this species [1,7,23,25,26]. Therefore, this study is one of the few to use clade-specific molecular markers to investigate the diversity of *M. africanum *strains causing TB within a specific African locale. Previous MTC species surveys that characterized strains using truly informative phylogenetic markers identified *M. africanum *West African-1, but not West African-2, in Cameroon and Nigeria [41,42] or *M. africanum *West African-2, but not West African-1, in The Gambia [38,44] and Guinea-Bissau [23,45]. In contrast, with this study, we highlight the fact that both clades of *M. africanum *are contributing to the TB burden in Ghana [24]. However, because the Ghana MTC collection was not representative, the current study does not allow us to estimate the proportion of TB caused by the various MTC clades in this country. Such a systematic survey of MTC population structure in Ghana is currently in progress.

**Figure 3:**
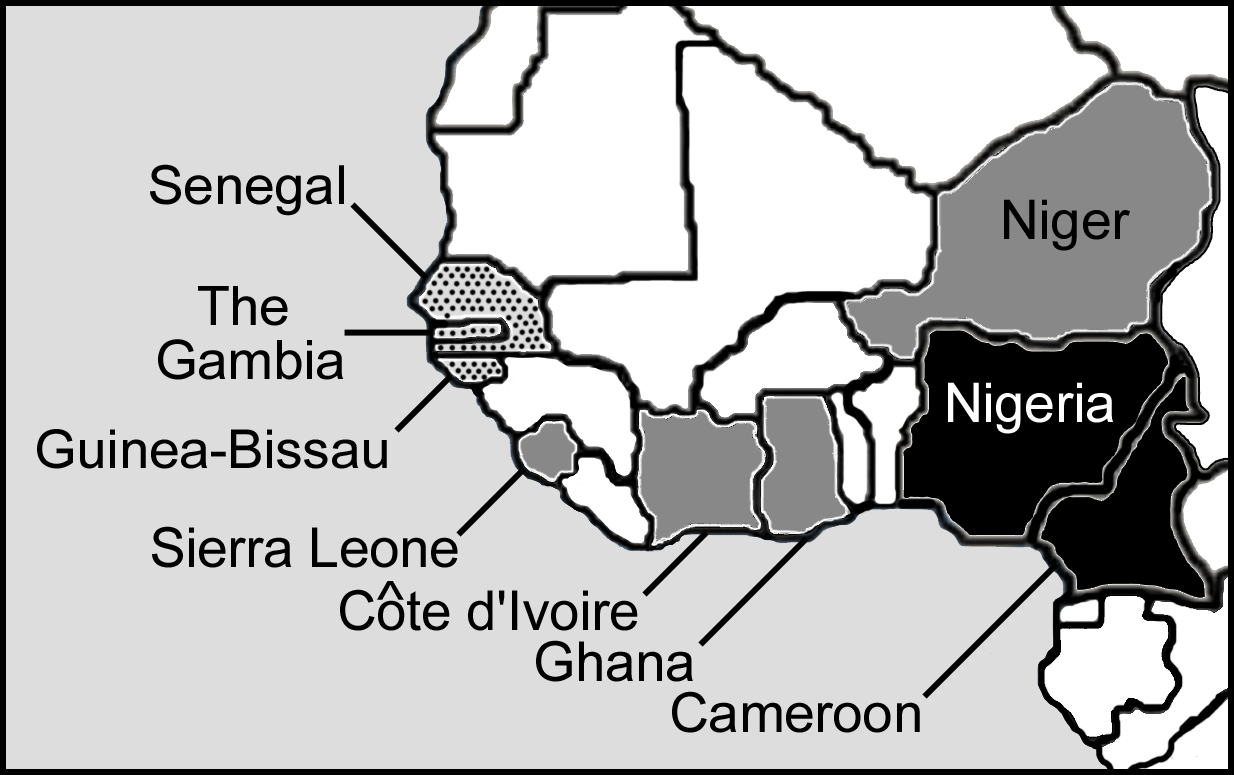
**Map of sub-Saharan West Africa illustrating the differential geographic distribution by country of the *M. africanum *clades**. Current evidence suggests that only *M. africanum *West African-1 is found in Eastern-West Africa (Cameroon, and Nigeria; black) and *M. africanum *West African-2 alone is found in Western-West Africa (the Gambia, Guinea-Bissau, and Senegal; speckled), but that the two clades overlap in Central-West Africa (Côte D'Ivoire, Ghana, Niger, and Sierra Leone; grey).

In actuality, few reports have definitively shown an overlap in the geographic ranges of *M. africanum *West African-1 and *M. africanum *West African-2. Previously, Huard *et al. *[7] studied isolates derived from patients in Niger that constituted both *M. africanum *clades; both lineages were likewise found to coexist in Sierra Leone [39]. In the absence of a molecular analysis similar to that presented herein, it is not known for certain which *M. africanum *clade predominates in many of the other *M. africanum*-endemic West African countries or if their ranges coincide elsewhere. However, a cross-comparison of molecular epidemiologic evidence presented in some earlier reports [17,46] and more recent data [7,41,43] does suggest that *M. africanum *clades 1 and 2 may both occur in at least Côte D'Ivoire, a country that borders Ghana. The picture that emerges from the combined studies [7,17,22-24,30,37-48] is of a differential geographic distribution of the *M. africanum *lineages, with West African-1 predominating in Eastern-West Africa (Cameroon, Nigeria), West African-2 in Western-West Africa (the Gambia, Guinea-Bissau, Senegal), and the two clades overlapping in Central-West Africa (Côte D'Ivoire, Ghana, Niger, Sierra Leone) (Fig. 3). A conceptually similar gradient of *M. africanum *prevalence across Western Africa was recently hypothesized by de Jong *et al.*, but their analysis did not make a distinction between the two *M. africanum *clades [48]. Lastly, although TB caused by *M. africanum *is concentrated in sub-Saharan West African countries, with immigration and international travel, sporadic cases have also been reported in the USA, the Caribbean, and Europe [28,43,49], including one outbreak of multi-drug resistant *M. africanum *at a Parisian hospital [17,50]. With improved molecular methods of identification, we expect that further cases of infection will be identified outside of the traditional endemic areas of *M. africanum*.

Molecular systems are preferred for the differentiation of *M. africanum *from *M. tuberculosis *and *M. bovis *given the heterogeneous phenotypic patterns among *M. africanum *strains, and the prolonged time-to-results and subjectivity inherent to the interpretation of some tests. Importantly, previous data indicate that there are no definitive phenotypic characteristics that can be exploited to differentiate the individual *M. africanum *clades [17,22,45]. In this study, we identified novel *M. africanum *clade-defining SNPs and confirmed the MTC distribution of several other phylogenetically relevant markers among the MTC. Multiple validated intra-species-specific molecular markers are important because they cross-corroborate each other and increase confidence in a given MTC species identification. By the markers described herein, *M. africanum *West African-1 would be defined genotypically as possessing RD713 and SNPs at *aroA*^285^, *Rv1332*^523^, and TbD1^197^, while *M. africanum *West African-2 would be defined genotypically by RD701 and RD702, as well as the intra-species-specific SNPs at *hsp*65^540 ^and *nat^751^*. Other SNPs and RDs that mark particular branches of the MTC phylogenetic tree, such as *gyr*B^1450^, *Rv1510*^1129^, RD9, and RD10 are also informative of *M. africanum *clade identity and provide further cross-referencing options. However, a streamlined protocol that employs 3-primer PCRs for RD9, RD10, and RD701 was the most rapid, simple, straight-forward and definitive means of differentiating the two clades of *M. africanum *from one another and from other MTC species. This approach limits the number of individual PCR reactions required for identification and eliminates the need for secondary procedures, such as restriction digestion, sequence analysis, or hybridization. Of note, some methods cannot distinguish the two clades of *M. africanum*, such as the GenoType MTBC line-probe assay [14-16]. Because PCR-RFA for SNPs specific to one of the *M. africanum *clades, as described herein, is a relatively simple approach, it may be of benefit for confirmation of species identification in laboratories with limited access to more advanced molecular methods. Other methods for *M. africanum *identification, such as by real-time PCR, microarray analysis, and spoligotyping (a DNA typing method) may also present advantages to laboratories with these capabilities, but these modalities were not evaluated in the current study.

Indeed, all strains of *M. africanum *are also known to lack spacers 9 and 39 in their spoligotype profile, similar to *M. bovis*, but possess one or more spacers that are consistently absent in certain other MTC species [7,25]. Previous data [17,23,37,46] suggest that many, but not all, *M. africanum *West African-1 strains demonstrate an absence of spacer 8 in addition to 9 and 39 (known as spoligotype signature AFRI_2) [43], while *M. africanum *West African-2 strains may further uniformly lack spacers 7-9 and 39 (known as spoligotype signature AFRI_1). As provided on the MIRU-VNTR*plus *website, all *M. africanum *West African-1 strains from the Ghana collection lacked spacers 8, 9, and 39, while each *M. africanum *West African-2 strain from the Ghana collection lacked spacers 7- 9, and 39 [27]. Spoligotyping may therefore provide a preliminary indicator for each *M. africanum *clade [51,52], however, the validity of these associations remains to be conclusively determined using a sample set of isolates with diverse geographical origins.

In addition to identification, MTC species and sub-lineage specific markers are of importance for genealogical purposes, as they allow the construction of more accurate phylogenetic trees. In recent years, SNP typing has been used to group strains of *M. tuberculosis *[53,54], while LSP analyses and DNA sequencing approaches have been used to establish congruent phylogenies for the *M. tuberculosis *complex [25,51,55]. The species- and sublineage-specific polymorphisms examined in this study for the *M. africanum *clades may therefore be of benefit when characterizing the evolutionary history of MTC strain sets in the future. SNPs in *rpoB*, for instance, demarcate the sequential divergence of sublineages within *M. africanum *West African-2 [7]. Similarly, we previously highlighted that RD711 is deleted in most, but not all of the RD713-harboring *M. africanum *West African-1 strains that were evaluated [7], and so defines a major sublineage within this species. (Studies that would use deletion of RD711 as the single marker to define *M. africanum *West Aftican-1 strains may therefore risk mis-categorizing some isolates.) Nonetheless, all the *M. africanum *West African-1 strains in the Ghana strain collection had RD711 deleted and, as part of another study [24], could be further subdivided phylogenetically based upon differences in mycobacterial tandem repeats numbers. Although not evaluated in this study, Mostowy *et al. *[23] recently reported that RD742 was also variably distributed among *M. africanum *West African-2 strains and a set of phylogenetically informative SNPs for *M. africanum*, different from those screened herein, has been published [51]. Overall, the combined data illustrate the continued evolutionary diversification of the *M. africanum *clades and advance the process of organizing a set of variable markers that may be used to construct meaningful phylogenetic trees for *M. africanum*. To this end, RD715 and RD743 were identified within *M. africanum *West African-1 strains [23] and single nucleotide changes located within the RD1 locus of *M. africanum *West African-2 strains were recently noted in select strains [38], but the utility of these polymorphisms as phylogenetic markers remains to be determined. It should also be mentioned that at least one *M. africanum*-like strain has been described with RD9 deleted, but RD7, RD10, RD702, RD711, and RD713 intact [56]. Combined, these data indicate that there is greater *M. africanum*/MTC diversity yet to be characterized.

Our understanding of the nature of *M. africanum *as a species and its position within the MTC has evolved considerably in recent years. Based upon hard genome level sequence evidence, the name *M. africanum *subtype II is no longer applied [2,7,20,22,23], while strains denoted as *M. africanum *subtype I are now, ironically, recognized to constitute two relatively genetically distinct lineages emerging from separate nodes along the MTC evolutionary tree [1,7,25,26]. This opinion is reinforced by the data provided in the current report. Interestingly, the above mentioned unique *M. africanum*-like strain was isolated from a patient originating from the Democratic Republic of Congo, a central African country [56]. As it has been postulated that the MTC originated near the horn of Africa [57], this strain may therefore be a remnant *M. africanum *precursor that evolved from *M. tuberculosis *as humans migrated from Eastern to Western Africa [55]. Indeed, the *M. africanum *clades possess the phenotypic and genotypic characteristics of sequential intermediary genotypes in the evolution of *M. bovis *from *M. tuberculosis *[1,7,24,26]. In so being, there have been suggestions that an *M. africanum *transmission cycle may exist between humans and an unknown animal reservoir [23]. Reports of *M. africanum *isolation from a bovine source in Nigeria and from a goat in Guinea Bissau support this hypothesis [37,42]. Therefore, a study of animal MTC isolates employing genetic markers, such as those we have organized herein, should be made a priority effort to rule out *M. africanum *as an important source of zoonotic and/or anthropozoonotic TB in Western Africa.

## Conclusions

With this study, we have organized a series of consistent phylogenetically-relevant markers for each of the distinct MTC lineages that share the *M. africanum *designation, highlighting those polymorphisms that can be used for specific clade identification. A review of molecular studies of *M. africanum *reveals a differential distribution of each *M. africanum *clade in Western Africa. Because *M. africanum *continues to be an important agent of disease, more *M. africanum*-focused studies are needed to increase our understanding of MTC pathobiology, epidemiology, and evolutionary history, all of which could lead to new strategies for TB prevention.

## Competing interests

The authors declare that they have no competing interests.

## Authors' contributions

SEGV and RCH: carried out the molecular genetic studies, participated in genotyping studies, analyzed the data and wrote the manuscript. SN: isolation and initial identification of the Ghana collection strains and provided suggestions during manuscript preparation. KK: isolation and identification of control strains and provided critical comments for the manuscript, ARS: provided methodological assistance and critical comments for the manuscript. RCH, PNS and JLH: conceived the study and the methodology and supervised the various stages of the research. PNS and JLH: coordinated the investigation and provided suggestions during manuscript preparation. All authors read and approved the final manuscript.

## Pre-publication history

The pre-publication history for this paper can be accessed here:

http://www.biomedcentral.com/1471-2334/10/80/prepub

## References

[B1] Brosch R Gordon SV Marmiesse M Brodin P Buchrieser C Eiglmeier K Garnier T Gutierrez C Hewinson G Kremer K Parsons LM Pym AS Samper S van Soolingen D Cole ST A new evolutionary scenario for the *Mycobacterium tuberculosis *complex Proc Natl Acad Sci USA 2002 99 6 3684 3689 10.1073/pnas.052548299 11891304 PMC122584

[B2] Huard RC Lazzarini LCO Butler W van Soolingen D Ho JL PCR-based method to differentiate the subspecies of the *Mycobacterium tuberculosis *complex on the basis of genomic deletions J Clin Microbiol 2003 41 4 1637 1650 10.1128/JCM.41.4.1637-1650.2003 12682155 PMC153936

[B3] Mostowy S Cousins D Brinkman J Aranaz A Behr M Genomic deletions suggest a phylogeny for the *Mycobacterium tuberculosis *complex J Infect Dis 2002 186 1 74 80 10.1086/341068 12089664

[B4] Aranaz A Cousins D Mateos A Domínguez L Elevation of *Mycobacterium tuberculosis subsp. caprae *Aranaz et al. 1999 to species rank as *Mycobacterium caprae comb. nov., sp. nov* Int J Syst Evol Microbiol 2003 53 6 1785 1789 10.1099/ijs.0.02532-0 14657105

[B5] Cousins D Bastida R Cataldi A Quse V Redrobe S Dow S Duignan P Murray A Dupont C Ahmed N Collins D Butler W Dawson D Rodríguez D Loureiro J Romano M Alito A Zumarraga M Bernardelli A Tuberculosis in seals caused by a novel member of the *Mycobacterium tuberculosis *complex: *Mycobacterium pinnipedii sp. nov* Int J Syst Evol Microbiol 2003 53 5 1305 1314 10.1099/ijs.0.02401-0 13130011

[B6] Cousins D Peet R Gaynor W Williams S Gow B Tuberculosis in imported hyrax (*Procavia capensis*) caused by an unusual variant belonging to the *Mycobacterium tuberculosis *complex Vet Microbiol 1994 42 2-3 135 145 10.1016/0378-1135(94)90013-2 7886928

[B7] Huard RC Fabre M de Haas P Lazzarini LCO van Soolingen D Cousins D Ho JL Novel genetic polymorphisms that further delineate the phylogeny of the *Mycobacterium tuberculosis *complex J Bacteriol 2006 188 12 4271 4287 10.1128/JB.01783-05 16740934 PMC1482959

[B8] van Soolingen D Hoogenboezem T de Haas P Hermans P Koedam M Teppema K Brennan P Besra G Portaels F Top J Schouls L van Embden J A novel pathogenic taxon of the *Mycobacterium tuberculosis *complex, Canetti: characterization of an exceptional isolate from Africa Int J Syst Bacteriol 1997 47 4 1236 1245 10.1099/00207713-47-4-1236 9336935

[B9] Scorpio A Zhang Y Mutations in *pncA*, a gene encoding pyrazinamidase/nicotinamidase, cause resistance to the antituberculous drug pyrazinamide in the tubercle bacillus Nat Med 1996 2 6 662 667 10.1038/nm0696-662 8640557

[B10] Niemann S Richter E Rüsch-Gerdes S Differentiation among members of the *Mycobacterium tuberculosis *complex by molecular and biochemical features: evidence for two pyrazinamide-susceptible subtypes of *M. bovis* J Clin Microbiol 2000 38 1 152 157 10618079 10.1128/jcm.38.1.152-157.2000 PMC86043

[B11] Goh K Legrand E Sola C Rastogi N Rapid differentiation of "*Mycobacterium canettii*" from other *Mycobacterium tuberculosis *complex organisms by PCR-restriction analysis of the *hsp65 *gene J Clin Microbiol 2001 39 10 3705 3708 10.1128/JCM.39.10.3705-3708.2001 11574597 PMC88413

[B12] Parsons L Brosch R Cole S Somoskövi A Loder A Bretzel G Van Soolingen D Hale Y Salfinger M Rapid and simple approach for identification of *Mycobacterium tuberculosis *complex isolates by PCR-based genomic deletion analysis J Clin Microbiol 2002 40 7 2339 2345 10.1128/JCM.40.7.2339-2345.2002 12089245 PMC120548

[B13] Warren R Gey van Pittius N Barnard M Hesseling A Engelke E de Kock M Gutierrez M Chege G Victor T Hoal E van Helden P Differentiation of *Mycobacterium tuberculosis *complex by PCR amplification of genomic regions of difference Int J Tuberc Lung Dis 2006 10 7 818 822 16850559

[B14] Richter E Weizenegger M Fahr A Rüsch-Gerdes S Usefulness of the GenoType MTBC assay for differentiating species of the *Mycobacterium tuberculosis *complex in cultures obtained from clinical specimens J Clin Microbiol 2004 42 9 4303 4306 10.1128/JCM.42.9.4303-4306.2004 15365028 PMC516283

[B15] Richter E Weizenegger M Rüsch-Gerdes S Niemann S Evaluation of GenoType MTBC assay for differentiation of clinical *Mycobacterium tuberculosis *complex isolates J Clin Microbiol 2003 41 6 2672 2675 10.1128/JCM.41.6.2672-2675.2003 12791901 PMC156502

[B16] Somoskovi A Dormandy J Rivenburg J Pedrosa M McBride M Salfinger M Direct comparison of the GenoType MTBC and genomic deletion assays in terms of ability to distinguish between members of the *Mycobacterium tuberculosis *Complex in clinical isolates and in clinical specimens J Clin Microbiol 2008 46 5 1854 1857 10.1128/JCM.00105-07 18353933 PMC2395102

[B17] Viana-Niero C Gutierrez C Sola C Filliol I Boulahbal F Vincent V Rastogi N Genetic diversity of *Mycobacterium africanum *clinical isolates based on IS*6110*-restriction fragment length polymorphism analysis, spoligotyping, and variable number of tandem DNA repeats J Clin Microbiol 2001 39 1 57 65 10.1128/JCM.39.1.57-65.2001 11136749 PMC87680

[B18] Haas W Bretzel G Amthor B Schilke K Krommes G Rüsch-Gerdes S Sticht-Groh V Bremer H Comparison of DNA fingerprint patterns of isolates of *Mycobacterium africanum *from east and west Africa J Clin Microbiol 1997 35 3 663 666 9041408 10.1128/jcm.35.3.663-666.1997 PMC229646

[B19] Niemann S Rüsch-Gerdes S Joloba M Whalen C Guwatudde D Ellner J Eisenach K Fumokong N Johnson J Aisu T Mugerwa R Okwera A Schwander S *Mycobacterium africanum *subtype II is associated with two distinct genotypes and is a major cause of human tuberculosis in Kampala, Uganda J Clin Microbiol 2002 40 9 3398 3405 10.1128/JCM.40.9.3398-3405.2002 12202584 PMC130701

[B20] Sola C Rastogi N Gutierrez M Vincent V Brosch R Parsons L Is *Mycobacterium africanum *subtype II (Uganda I and Uganda II) a genetically well-defined subspecies of the *Mycobacterium tuberculosis *complex? J Clin Microbiol 2003 41 3 1345 1346 10.1128/JCM.41.3.1345-1348.2003 12624085 PMC150321

[B21] Asiimwe B Koivula T Källenius G Huard R Ghebremichael S Asiimwe J Joloba M *Mycobacterium tuberculosis *Uganda genotype is the predominant cause of TB in Kampala, Uganda Int J Tuberc Lung Dis 2008 12 4 386 391 18371263

[B22] Niemann S Kubica T Bange F Adjei O Browne E Chinbuah M Diel R Gyapong J Horstmann R Joloba M Meyer C Mugerwa R Okwera A Osei I Owusu-Darbo E Schwander S Rüsch-Gerdes S The species *Mycobacterium africanum *in the light of new molecular markers J Clin Microbiol 2004 42 9 3958 3962 10.1128/JCM.42.9.3958-3962.2004 15364975 PMC516319

[B23] Mostowy S Onipede A Gagneux S Niemann S Kremer K Desmond E Kato-Maeda M Behr M Genomic analysis distinguishes *Mycobacterium africanum* J Clin Microbiol 2004 42 8 3594 3599 10.1128/JCM.42.8.3594-3599.2004 15297503 PMC497617

[B24] Wirth T Hildebrand F Allix-Béguec C Wölbeling F Kubica T Kremer K van Soolingen D Rüsch-Gerdes S Locht C Brisse S Meyer A Supply P Niemann S Origin, spread and demography of the *Mycobacterium tuberculosis *complex PLoS Pathog 2008 4 9 e1000160 10.1371/journal.ppat.1000160 18802459 PMC2528947

[B25] Gagneux S DeRiemer K Van T Kato-Maeda M de Jong B Narayanan S Nicol M Niemann S Kremer K Gutierrez M Hilty M Hopewell P Small P Variable host-pathogen compatibility in *Mycobacterium tuberculosis* Proc Natl Acad Sci USA 2006 103 8 2869 2873 10.1073/pnas.0511240103 16477032 PMC1413851

[B26] Smith N Kremer K Inwald J Dale J Driscoll J Gordon S van Soolingen D Hewinson R Smith J Ecotypes of the *Mycobacterium tuberculosis *complex J Theor Biol 2006 239 2 220 225 10.1016/j.jtbi.2005.08.036 16242724

[B27] Allix-Béguec C Harmsen D Weniger T Supply P Niemann S Evaluation and strategy for use of MIRU-VNTR*plus*, a multifunctional database for online analysis of genotyping data and phylogenetic identification of *Mycobacterium tuberculosis *complex isolates J Clin Microbiol 2008 46 8 2692 2699 10.1128/JCM.00540-08 18550737 PMC2519508

[B28] Kremer K van Soolingen D Frothingham R Haas W Hermans P Martín C Palittapongarnpim P Plikaytis B Riley L Yakrus M Musser J van Embden J Comparison of methods based on different molecular epidemiological markers for typing of *Mycobacterium tuberculosis *complex strains: interlaboratory study of discriminatory power and reproducibility J Clin Microbiol 1999 37 8 2607 2618 10405410 10.1128/jcm.37.8.2607-2618.1999 PMC85295

[B29] Sreevatsan S Pan X Stockbauer K Connell N Kreiswirth B Whittam T Musser J Restricted structural gene polymorphism in the *Mycobacterium tuberculosis *complex indicates evolutionarily recent global dissemination Proc Natl Acad Sci USA 1997 94 18 9869 9874 10.1073/pnas.94.18.9869 9275218 PMC23284

[B30] Frothingham R Strickland P Bretzel G Ramaswamy S Musser J Williams D Phenotypic and genotypic characterization of *Mycobacterium africanum *isolates from West Africa J Clin Microbiol 1999 37 6 1921 1926 10325347 10.1128/jcm.37.6.1921-1926.1999 PMC84985

[B31] Lazzarini LCO Huard RC Boechat N Gomes H Oelemann M Kurepina N Shashkina E Mello F Gibson A Virginio M Marsico A Butler W Kreiswirth B Suffys P Lapa E Silva JR Ho JL Discovery of a novel *Mycobacterium tuberculosis *lineage that is a major cause of tuberculosis in Rio de Janeiro, Brazil J Clin Microbiol 2007 45 12 3891 3902 10.1128/JCM.01394-07 17898156 PMC2168543

[B32] Gibson A Huard R Gey van Pittius N Lazzarini L Driscoll J Kurepina N Zozio T Sola C Spindola S Kritski A Fitzgerald D Kremer K Mardassi H Chitale P Brinkworth J Garcia de Viedma D Gicquel B Pape J van Soolingen D Kreiswirth B Warren R van Helden P Rastogi N Suffys P Lapa e Silva J Ho J Application of sensitive and specific molecular methods to uncover global dissemination of the major RD^Rio ^Sublineage of the Latin American-Mediterranean *Mycobacterium tuberculosis *spoligotype family J Clin Microbiol 2008 46 4 1259 1267 10.1128/JCM.02231-07 18234868 PMC2292928

[B33] Goh K Rastogi N Berchel M Huard R Sola C Molecular evolutionary history of tubercle bacilli assessed by study of the polymorphic nucleotide within the nitrate reductase (*narGHJI*) operon promoter J Clin Microbiol 2005 43 8 4010 4014 10.1128/JCM.43.8.4010-4014.2005 16081943 PMC1233921

[B34] Constant P Perez E Malaga W Laneelle MA Saurel O Daffe M Guilhot C Role of the *pks15/1 *gene in the biosynthesis of phenolglycolipids in the *Mycobacterium tuberculosis *complex - Evidence that all strains synthesize glycosylated p-hydroxybenzoic methyl esters and that strains devoid of phenolglycolipids harbor a frameshift mutation in the *pks15/1 *gene Journal of Biological Chemistry 2002 277 41 38148 38158 10.1074/jbc.M206538200 12138124

[B35] Huard R Chitale S Leung M Lazzarini L Zhu H Shashkina E Laal S Conde M Kritski A Belisle J Kreiswirth B Lapa e Silva J Ho J The *Mycobacterium tuberculosis *complex-restricted gene *cfp32 *encodes an expressed protein that is detectable in tuberculosis patients and is positively correlated with pulmonary interleukin-10 Infect Immun 2003 71 12 6871 6883 10.1128/IAI.71.12.6871-6883.2003 14638775 PMC308900

[B36] Sholto-Douglas-Vernon C Sandy J Victor T Sim E Helden P Mutational and expression analysis of *tbnat *and its response to isoniazid J Med Microbiol 2005 54 12 1189 1197 10.1099/jmm.0.46153-0 16278433

[B37] Källenius G Koivula T Ghebremichael S Hoffner S Norberg R Svensson E Dias F Marklund B Svenson S Evolution and clonal traits of *Mycobacterium tuberculosis *complex in Guinea-Bissau J Clin Microbiol 1999 37 12 3872 3878 10565899 10.1128/jcm.37.12.3872-3878.1999 PMC85833

[B38] de Jong B Hill P Brookes R Gagneux S Jeffries D Otu J Donkor S Fox A McAdam K Small P Adegbola R *Mycobacterium africanum *elicits an attenuated T cell response to early secreted antigenic target, 6 kDa, in patients with tuberculosis and their household contacts J Infect Dis 2006 193 9 1279 1286 10.1086/502977 16586366

[B39] Homolka S Post E Oberhauser B George A Westman L Dafae F Rüsch-Gerdes S Niemann S High genetic diversity among *Mycobacterium tuberculosis *complex strains from Sierra Leone BMC Microbiol 2008 8 103 10.1186/1471-2180-8-103 18578864 PMC2447842

[B40] Ledru S Cauchoix B Yaméogo M Zoubga A Lamandé-Chiron J Portaels F Chiron J Impact of short-course therapy on tuberculosis drug resistance in South-West Burkina Faso Tuber Lung Dis 1996 77 5 429 436 10.1016/S0962-8479(96)90116-1 8959147

[B41] Niobe-Eyangoh S Kuaban C Sorlin P Cunin P Thonnon J Sola C Rastogi N Vincent V Gutierrez M Genetic biodiversity of *Mycobacterium tuberculosis *complex strains from patients with pulmonary tuberculosis in Cameroon J Clin Microbiol 2003 41 6 2547 2553 10.1128/JCM.41.6.2547-2553.2003 12791879 PMC156567

[B42] Cadmus S Palmer S Okker M Dale J Gover K Smith N Jahans K Hewinson R Gordon S Molecular analysis of human and bovine tubercle bacilli from a local setting in Nigeria J Clin Microbiol 2006 44 1 29 34 10.1128/JCM.44.1.29-34.2006 16390943 PMC1351927

[B43] Brudey K Driscoll J Rigouts L Prodinger W Gori A Al-Hajoj S Allix C Aristimuño L Arora J Baumanis V Binder L Cafrune P Cataldi A Cheong S Diel R Ellermeier C Evans J Fauville-Dufaux M Ferdinand S Garcia de Viedma D Garzelli C Gazzola L Gomes H Guttierez M Hawkey P van Helden P Kadival G Kreiswirth B Kremer K Kubin M Kulkarni S Liens B Lillebaek T Ho M Martin C Mokrousov I Narvskaïa O Ngeow Y Naumann L Niemann S Parwati I Rahim Z Rasolofo-Razanamparany V Rasolonavalona T Rossetti M Rüsch-Gerdes S Sajduda A Samper S Shemyakin I Singh U Somoskovi A Skuce R van Soolingen D Streicher E Suffys P Tortoli E Tracevska T Vincent V Victor T Warren R Yap S Zaman K Portaels F Rastogi N Sola C Mining the fourth international spoligotyping database (SpolDB4) for classification, population genetics and epidemiology BMC Microbiol 2006 6 23 10.1186/1471-2180-6-23 16519816 PMC1468417

[B44] de Jong B Hill P Aiken A Jeffries D Onipede A Small P Adegbola R Corrah T Clinical presentation and outcome of tuberculosis patients infected by *M. africanum *versus *M. tuberculosis* Int J Tuberc Lung Dis 2007 11 4 450 456 17394693

[B45] Koivula T Ekman M Leitner T Löfdahl S Ghebremicahel S Mostowy S Behr M Svenson S Källenius G Genetic characterization of the Guinea-Bissau family of *Mycobacterium tuberculosis *complex strains Microbes Infect 2004 6 3 272 278 10.1016/j.micinf.2003.12.006 15026014

[B46] Brudey K Gutierrez M Vincent V Parsons L Salfinger M Rastogi N Sola C *Mycobacterium africanum *genotyping using novel spacer oligonucleotides in the direct repeat locus J Clin Microbiol 2004 42 11 5053 5057 10.1128/JCM.42.11.5053-5057.2004 15528695 PMC525283

[B47] Niang M de la Salmoniere Y Samb A Hane A Cisse M Gicquel B Perraut R Characterization of *M. tuberculosis *strains from west African patients by spoligotyping Microbes Infect 1999 1 14 1189 1192 10.1016/S1286-4579(99)00243-9 10580274

[B48] de Jong B Antonio M Awine T Ogungbemi K de Jong Y Gagneux S De Riemer K Zozio T Rastogi N Borgdorff M Hill P Adegbola R Use of spoligotyping and large sequence polymorphisms to study the population structure of the *Mycobacterium tuberculosis *complex in a cohort study of consecutive smear-positive tuberculosis cases in The Gambia J Clin Microbiol 2009 47 4 994 1001 10.1128/JCM.01216-08 19193842 PMC2668362

[B49] Desmond E Ahmed A Probert W Ely J Jang Y Sanders C Lin S Flood J *Mycobacterium africanum *cases, California Emerg Infect Dis 2004 10 5 921 923 15200832 10.3201/eid1005.030016

[B50] Gutiérrez M Galán J Blázquez J Bouvet E Vincent V Molecular markers demonstrate that the first described multidrug-resistant *Mycobacterium bovis *outbreak was due to *Mycobacterium tuberculosis* J Clin Microbiol 1999 37 4 971 975 10074511 10.1128/jcm.37.4.971-975.1999 PMC88634

[B51] Comas I Homolka S Niemann S Gagneux S Genotyping of genetically monomorphic bacteria: DNA sequencing in *Mycobacterium tuberculosis *highlights the limitations of current methodologies PLoS ONE 2009 4 11 e7815 10.1371/journal.pone.0007815 19915672 PMC2772813

[B52] Gagneux S Small P Global phylogeography of *Mycobacterium tuberculosis *and implications for tuberculosis product development Lancet Infect Dis 2007 7 5 328 337 10.1016/S1473-3099(07)70108-1 17448936

[B53] Filliol I Motiwala A Cavatore M Qi W Hazbón M Bobadilla del Valle M Fyfe J García-García L Rastogi N Sola C Zozio T Guerrero M León C Crabtree J Angiuoli S Eisenach K Durmaz R Joloba M Rendón A Sifuentes-Osornio J Ponce de León A Cave M Fleischmann R Whittam T Alland D Global phylogeny of *Mycobacterium tuberculosis *based on single nucleotide polymorphism (SNP) analysis: insights into tuberculosis evolution, phylogenetic accuracy of other DNA fingerprinting systems, and recommendations for a minimal standard SNP set J Bacteriol 2006 188 2 759 772 10.1128/JB.188.2.759-772.2006 16385065 PMC1347298

[B54] Gutacker M Smoot J Migliaccio C Ricklefs S Hua S Cousins D Graviss E Shashkina E Kreiswirth B Musser J Genome-wide analysis of synonymous single nucleotide polymorphisms in *Mycobacterium tuberculosis *complex organisms: resolution of genetic relationships among closely related microbial strains Genetics 2002 162 4 1533 1543 12524330 10.1093/genetics/162.4.1533 PMC1462380

[B55] Hershberg R Lipatov M Small PM Sheffer H Niemann S Homolka S Roach JC Kremer K Petrov DA Feldman MW Gagneux S High functional diversity in *Mycobacterium tuberculosis *driven by genetic drift and human demography PLoS Biol 2008 6 12 e311 10.1371/journal.pbio.0060311 19090620 PMC2602723

[B56] Reed M Pichler V McIntosh F Mattia A Fallow A Masala S Domenech P Zwerling A Thibert L Menzies D Schwartzman K Behr M Major *Mycobacterium tuberculosis *lineages associate with patient country of origin J Clin Microbiol 2009 47 4 1119 1128 10.1128/JCM.02142-08 19213699 PMC2668307

[B57] Gutierrez MC Brisse S Brosch R Fabre M Omaïs B Marmiesse M Supply P Vincent V Ancient origin and gene mosaicism of the progenitor of *Mycobacterium tuberculosis* PLoS Pathog 2005 1 1 e5 10.1371/journal.ppat.0010005 16201017 PMC1238740

